# Novel melanoma therapy

**DOI:** 10.1186/s40164-016-0054-1

**Published:** 2016-08-08

**Authors:** Eddy C. Hsueh, Kalyan C. Gorantla

**Affiliations:** Division of General Surgery, Department of Surgery, Saint Louis University, 3635 Vista at Grand Blvd, St. Louis, MO 63110 USA

**Keywords:** Metastatic melanoma, Checkpoint inhibitors, Targeted therapy

## Abstract

With the rapid succession of new effective agents for melanoma in the recent years, the paradigm for treatment of metastatic melanoma is changing. The success of combining multiple effective agents compared with outcomes of monotherapy also brings increasing complexity in the treatment algorithm for various subsets of metastatic melanoma patients. We reviewed the recent reports on novel melanoma therapy to shed light on rational decision-making in treating these patients.

## Background

Recent rapid succession of breakthroughs in melanoma therapy has dramatically changed the treatment paradigm for metastatic melanoma over the last 5 years. The remarkable success of immune checkpoint inhibitors in metastatic melanoma treatment has validated immunotherapy as a major modality for cancer treatment in addition to surgery, chemotherapy, and radiation therapy. The elucidation of molecular pathways in cancer biology has given rise to targeted therapy with dramatic responses in select metastatic melanoma patients. The development of oncolytic virus in melanoma treatment has brought forth the first FDA-approved oncolytic viral therapy for cancer. With these exciting breakthroughs in melanoma therapy and continuing to emerge novel therapies for melanoma, the landscape of therapeutic options for patients with advanced stages of melanoma has drastically changed in comparison to options available for 30 plus years until 2011. In 2011, the FDA approved ipilimumab, a cytotoxic T-lymphocyte 4 (CTLA-4) inhibitor, based on the results of a 3-arm randomized phase III trial comparing a gp100 vaccine alone, ipilimumab alone, and the combination of vaccine with ipilimumab [1]. Subsequently, vemurafenib, a BRAF inhibitor, was approved in the same year for patients with metastatic melanoma harboring BRAF mutation [2, 3]. In 2013, the combination of BRAF inhibitor, dabrafenib, and MEKinhibitor, trametinib, was approved for BRAF mutation positive metastatic melanoma patients [4]. Approval of two inhibitors of the programmed death 1 (PD-1) receptor, pembrolizumab and nivolumab, were granted in 2014 [5, 6]. This review will attempt to summarize the advancement in novel melanoma therapy in the literature from the recent years and provide a reference for treatment decision consideration.

## Targeted therapy

Mutation in the serine-threonine kinase BRAF was observed in close to 50 % of metastatic melanoma lesions, mostly of the valine to glutamine substitution in codon 600 (V600E) [7]. Other less common BRAF mutations with lysine (V600K) or arginine (V600R) substitutions have also been reported. Vemurafenib and dabrafenib are the currently FDA-approved BRAF inhibitors that target these mutations for the treatment of melanoma. BRAF inhibitors have shown high rates of response (48–59 %) in phase II and III trials but with limited duration [2, 3, 8]. The median progression-free survival (PFS) ranges from 5.1 to 6.8 months [8, 9]. The lack of durability of response is due to the development of resistance, mainly reactivation of the mitogen-activated protein kinase (MAPK) pathway. Although reactivation of MAPK pathway was observed in over two-thirds of BRAF-inhibitor progressing tumors, amplification of parallel signaling networks such as the PI3K–PTEN–AKT pathway were also found [10–12]. Based on preclinical models, combined treatment with BRAF and MEK inhibitors might attenuate development of resistance by blocking reactivation of the MAPK pathway induced by single-agent BRAF inhibitors [13, 14]. As a first-line monotherapy, treatment with MEK inhibitor trametinib has also shown similar response rate (48 %) and PFS (4.8 months) compared with BRAF inhibitors [15].

Combination of BRAF/MEK inhibition with dabrafenib (BRAF inhibitor) and trametinib (MEK inhibitor) was evaluated in a phase I/II study [4]. The absence of drug–drug interaction was confirmed in 8 patients with repeated doses of trametinib and a single dose of dabrafenib. Escalating doses of dabrafenib (75 and 150 mg twice daily [BID]) in combination with trametinib (1, 1.5 and 2 mg every day [QD]) were evaluated in 77 patients to determine toxicity profile and pharmacokinetic activity. In the phase II expansion study, 162 advanced melanoma patients with BRAFV600 mutation and no prior BRAF targeted therapy were assigned 1:1:1 to receive dabrafenib (150 mg QD) and trametinib (either 1 or 2 mg QD) or dabrafenib (150 mg QD) monotherapy. Median PFS for those in the combination 150/2 group was 9.4 versus 5.8 months for patients who received dabrafenib monotherapy (hazard ratio [HR] for progression or death, 0.39; p < 0.001). In a subsequent phase III study (COMBI-d), 423 previously untreated and unresectable stage IIIC or stage IV melanoma patients with BRAF V600E or V600K mutation were randomized to receive combination of dabrafenib (150 mg BID) and trametinib (2 mg QD) or dabrafenib and placebo [16]. With a median follow-up of 9 months, median PFS was 9.3 months in combination group and 8.8 months in dabrafenib-only group (HR 0.75; 95 % Confidence Interval [CI] 0.57–0.99; p = 0.03). Similar rates of adverse events (AEs) were observed between the two groups. However, the rate of cutaneous squamous-cell carcinoma was lower in the combination group compared with dabrafenib-only group (2 versus 9 %). Pyrexia were more common (51 versus 28 %) and with more severity (grade 3, 6 versus 2 %) in the combination group. In a follow-up report, the median overall survival (OS) was 25.1 months in the combination group versus 18.7 months in the dabrafenib only group (HR 0.71, 95 % CI 0.55–0.92; p = 0.0107) [17]. The median PFS was 11.0 months in the combination group and 8.8 months in the dabrafenib only group (HR 0.67, 95 % CI 0.53–0.84; p = 0.0004). In a second phase III trial, combination of dabrafenib (150 mg BID) and trametinib (2 mg QD) were compared with vemurafenib (960 mg BID) orally as first-line therapy (COMBI-v) [18]. The combination also improved OS, PFS, and overall response rates (ORR) compared with vemurafenib alone group. The OS rate at 12 months was 72 % in the combination group and 65 % in the vemurafenib group (HR 0.69; 95 % CI 0.53–0.89; p = 0.005). Median PFS was 11.4 months in the combination group and 7.3 months in the vemurafenib group (HR 0.56; 95 % CI, 0.46–0.69; p < 0.001). ORR rate was 64 % in the combination group and 51 % in the vemurafenib group (p < 0.001). Cutaneous squamous-cell carcinoma and keratoacanthoma occurred in 1 % of patients in the combination group and 18 % of those in the vemurafenib group. In all three studies, rates of AEs were similar between the combination group and the monotherapy group. However, AEs related to reactivation of the MAPK pathway were reduced in the combination group compared with BRAF inhibitor monotherapy group. Pyrexia was more common and more severe in the combination group.

Combination therapy with BRAF inhibitor vemurafenib and MEK inhibitor cobimetinib were also evaluated in clinical trials with similar ORR, PFS and OS as dabrafenib and trametinib combination. In the phase Ib BRIM7 trial, patients who progressed on vemurafenib (n = 66) or never received BRAF inhibitor (n = 63) were enrolled and treated on ten dosing regimens with vemurafenib 720 or 960 mg BID continuously and cobimetinib 60, 80, or 100 mg QD for either 14 days on and 14 days off, 21 days on and 7 days off, or continuously [19]. Dose-limiting toxic effects were observed in four patients: grade 3 fatigue for more than 7 days, grade 3 prolongation of QTc, grade 3 stomatitis and fatigue, and arthralgia and myalgia. The maximum tolerated dose was established as vemurafenib 960 mg BID in combination with cobimetinib 60 mg QD 21 days on and 7 days off. Confirmed objective responses were observed in 10 of 66 patients (15 %) who had recently progressed on vemurafenib, with a median PFS of 2.8 months. Confirmed objective responses were noted in 55 of 63 BRAF inhibitor naïve patients (87 %) with median PFS of 13.7 months. All 63 patients who had never received a BRAF inhibitor had evidence of target lesion reduction after combination treatment. Recent update to the study was reported with additional 11 months of follow up [20]. Median OS of 28.5 months in the vemurafenib-naïve and 8.4 months in the vemurafenib-progressing patients was reported, with 2-year OS rates of 61.1 and 15.1 % respectively. In the subsequent phase III coBRIM trial, 495 patients with treatment-naïve BRAF V600 mutation–positive melanoma were randomized to receive vemurafenib (960 mg BID) and cobimetinib (60 mg QD 21 days on and 7 days off) (combination group) or vemurafenib and placebo (control group) [21]. Median PFS was 9.9 months in the combination group and 6.2 months in the control group (HR 0.51; 95 % CI 0.39–0.68; p < 0.001). ORR was 68 % in the combination group as compared with 45 % in the control group (p < 0.001), with 10 % complete response (CR) in the combination group and 4 % CR in the control group. The combination treatment was associated with higher incidence of grade 3 or higher AEs, as compared with control (65 versus 59 %). The incidence of secondary cutaneous squamous cell cancer was also decreased in the combination group compared with control group (2 versus 11 %). Recent update with 8 months additional follow up reported a PFS of 12.3 months in the combination group compared with 7.3 months for the control group (HR 0.58) and ORR of 70 versus 50 %, respectively [22].

Another BRAF and MEK inhibitor combination in clinical trial is encorafenib and binimetinib. In a phase Ib/II trial patients with advanced BRAFV600 positive melanoma were treated with 400, 450 or 600 mg QD of encorafenib and 45 mg BID of binimetinib [23]. ORR of 74.5 % with a median PFS of 11.3 months (95 % CI 7.4–14.6) were observed in the BRAF inhibitor naïve patients (n = 55). A 3-arm phase III trial (COLUMBUS) is ongoing where patients are randomized 1:1:1 to encorafenib 450 mg QD and binimetinib 45 mg BID, encorafenib alone at 300 mg QD, and vemurafenib alone [ClinicalTrials.gov identifier: NCT01909453].

Recently, encouraging results were also reported with agent that targets NRAS mutant melanomas in an open label phase III trial randomizing NRAS-mutant melanoma patients with or without prior immunotherapy 2:1 to binimetinib 45 mg BID (n = 269) or DTIC 1000 mg/m^2^ every 3 weeks (n = 133) [24]. Confirmed ORR and disease control rate (DCR) were 15 and 58 % for binimetinib group compared with 7 and 25 % for DTIC group (p = 0.015 [ORR]; p < 0.001 [DCR]), respectively. Median duration of response on binimetinib was 6.9 months.

The studies reviewed above are briefly summarized in Table 1.

**Table 1:** Recent targeted therapy study results in melanoma

Study	Agent(s)	Phase of study	Median PFS (months)	OS	ORR (%)
[4]	Dabrafenib + trametinib	II	9.4	79 % at 1 year	76
	Dabrafenib		5.8	70 % at 1 year	54
[16] [17]	Dabrafenib + trametinib	III	11.0	25.1 months	67
	Dabrafenib		8.8	18.7 months	51
[18]	Dabrafenib + trametinib	III	11.4	72 % at 1 year	64
	Vemurafenib		7.3	65 % at 1 year	51
[19] [20]	Cobimetinib + vemurafenib	I/II	Vemurafenib refractory: 2.8	8.4 months	15
			Vemurafenib naïve: 13.7	28.5 months	87
[21] [22]	Cobimetinib + vemurafenib	III	12.3	81 % at 9 months	70
	Vemurafenib		7.3	73 % at 9 months	50
[23]	Encorafenib + binimetinib	I/II	11.3	NA	74.5
[24]	Binimetinib	III	NA	NA	15
	DTIC				7

## Checkpoint inhibitors

Pembrolizumab, the first anti–PD-1 antibody to be approved by the FDA, is a highly selective, humanized monoclonal IgG4-kappa isotype antibody against PD-1. The results of the first phase I trial led to the FDA approval of pembrolizumab in ipilimumab-refractory metastatic melanoma patients in September 2014 [5, 25]. Patients with advanced melanoma with or without prior ipilimumab therapy (n = 135) were enrolled (KEYNOTE-001) [25]. Doses of pembrolizumab at either 2 or 10 mg/kg given every 2 or 3 weeks were tested. ORR of 31–51 and 81 % 1 year survival were observed. To address the utility of pembrolizumab in ipilimumab refractory patient population, an expansion cohort of 173 patients was enrolled in this phase I study [5]. Patients with advanced melanoma whose disease had progressed after ≥2 ipilimumab doses were randomly assigned to 2 mg/kg pembrolizumab every 3 weeks (n = 89) or 10 mg/kg every 3 weeks (n = 84). ORR was 26 % at both doses. The drug was well tolerated at both doses with similar safety profile and no drug-related deaths. Majority of patients in both doses had reduction of the target lesion size from baseline: 59 (73 %) patients in the pembrolizumab 2 mg/kg group and 52 (68 %) in the 10 mg/kg group. The most common drug-related AEs of any grade in the 2 mg/kg and 10 mg/kg groups were fatigue (33 versus 37 %), pruritus (26 versus 19 %), and rash (18 versus 18 %). Further evaluation of pembrolizumab was conducted comparing with investigator-choice chemotherapy in ipilimumab-refractory patients (KEYNOTE-002) [26]. In a randomized phase 2 trial enrolling patients with progressive disease after ≥2 ipilimumab doses 540 patients were randomly assigned (1:1:1) to receive pembrolizumab 2 mg/kg or 10 mg/kg every 3 weeks or investigator-choice chemotherapy (paclitaxel plus carboplatin, paclitaxel, carboplatin, dacarbazine, or oral temozolomide). PFS was improved in pembrolizumab 2 mg/kg cohort (HR 0.57, 95 % CI 0.45–0.73; p < 0.0001) and pembrolizumab 10 mg/kg cohort (0.50, 0.39–0.64; p < 0.0001) compared with chemotherapy group. PFS rates at 6 months were 34 % in pembrolizumab 2 mg/kg group, 38 % in the 10 mg/kg group, and 16 % in the chemotherapy group. Treatment-related grade 3–4 AEs were less in pembolizumab treatment groups, 11 % for 2 mg/kg group and 14 % for 10 mg/kg group, compared with 26 % in the chemotherapy group. Again noted in the pembrolizumab groups, the most common treatment-related grade 3–4 AE was fatigue (1 % in the 2 mg/kg group and <1 % in the 10 mg/kg group, compared with 5 % in the chemotherapy group). A follow-up pooled analysis of the 655 enrolled KEYNOTE-001 patients (135 from a nonrandomized cohort [n = 87 ipilimumab naive; n = 48 ipilimumab treated] and 520 from randomized cohorts [n = 226 ipilimumab naive; n = 294 ipilimumab treated]) was reported in 2016 [27]. Among the 581 patients with measurable disease at baseline, ORR was 33 with 45 % in treatment-naive patients. 44 % (90/205) of patients had response duration for at least 1 year and 79 % (162/205) had response duration for at least 6 months. Median OS in the total population was 23 months with a 24-month survival rate of 49 %. In treatment-naive patients, median OS was 31 months with a 24-month survival rate of 60 %. It was further noted that a smaller tumor size at baseline was associated with a higher ORR. There was no significant difference in antitumor activity among the various pembrolizumab doses in randomized cohorts. The study results were recently updated to include 3 year overall survival data [28]. The 36-month OS rate was 40 % and median OS was 23.8 months. Based on the finding of significant activity of pembrolizumab in treatment naïve patients, a randomized controlled phase III study was performed (KEYNOTE 006) enrolling 834 patients with advanced melanoma assigned in a 1:1:1 ratio to pembrolizumab 10 mg/kg every 2 weeks or every 3 weeks or four doses of ipilimumab 3 mg/kg every 3 weeks [29]. The estimated 6-month PFS rates were 47.3 % for pembrolizumab every 2 weeks, 46.4 % for pembrolizumab every 3 weeks, and 26.5 % for ipilimumab (HR 0.58; p < 0.001 for both pembrolizumab regimens versus ipilimumab; CIs 0.46–0.72 and 0.47–0.72, respectively). Estimated 12-month survival rates were 74.1, 68.4, and 58.2 %, respectively (HR for pembrolizumab every 2 weeks, 0.63; 95 % CI 0.47–0.83; p = 0.0005; HR for pembrolizumab every 3 weeks, 0.69; 95 % CI 0.52–0.90; p = 0.0036). Significant improvement in ORR were observed in both pembrolizumab treatment groups (33.7 % for every 2 weeks and 32.9 % for every 3 weeks) compared with ipilimumab group (11.9 %) (p < 0.001 for both comparisons). Reduced rates of treatment-related AEs of grade 3 to 5 severity were noted in the pembrolizumab groups (13.3 and 10.1 %) compared with ipilimumab group (19.9 %). Based on the fewer toxicities and significantly improved OS in the pembrolizumab treatment groups compared with ipilimumab group, FDA approved pembrolizumab as first-line therapy for metastatic melanoma. At a recent update with median follow-up duration of 22.9 months, the improvement in OS, PFS, and ORR with pembrolizumab over ipilimumab held up [30]. Median OS was not reached for either pembrolizumab groups versus 16.0 months with ipilimumab. The estimated 24-month OS rates were 55 % for pembrolizumab group and 43 % for ipilimumab group.

Nivolumab, a fully human IgG4 monoclonal antibody, was the first anti–PD-1 antibody to be evaluated in humans in a phase I trial [31]. Nivolumab at doses of 0.1–10 mg/kg produced a 31 % response rate in 107 previously treated ipilimumab-naive metastatic melanoma patients in a phase I trial [6]. Median duration of response and median OS were 22 months and 17.3 months, respectively. Two subsequent randomized trials comparing nivolumab with chemotherapy have been reported in ipilimumab refractory patients and in untreated patients without BRAF mutation [32, 33]. Metastatic melanoma patients without BRAF mutation (n = 418) were randomized to nivolumab 3 mg/kg every 2 weeks or dacarbazine 1000 mg/m^2^ every 3 weeks as first line therapy [32]. Compared with dacarbazine as first-line therapy, nivolumab improved 1-year OS rate, (72.9 versus 42.1 %; HR 0.42; 99.79 % CI, 0.25–0.73; p < 0.001), median PFS (5.1 versus 2.2 months; HR 0.43; 95 % CI 0.34–0.56; p < 0.001) and ORR (40.0 versus 13.9 %; odds ratio 4.06; p < 0.001). Rates of common AEs associated with nivolumab such as fatigue, pruritus, and nausea were consistent with findings from phase I trials. Grade 3 or 4 AEs occurred in 11.7 % of the nivolumab group and 17.6 % of the dacarbazine group.

For ipilimumab refractory patients, nivolumab was also found to be superior to chemotherapy [33]. Patients who progressed after ipilimumab or ipilimumab and a BRAF inhibitor if they were BRAF V600 mutation-positive were randomized 2:1 to nivolumab 3 mg/kg every 2 weeks (n = 272) or investigator choice chemotherapy (n = 133). An ORR of 31.7 % was observed in the nivolumab group compared with 10.6 % in the chemotherapy group. There was no significant difference in response to nivolumab in patients with or without previous benefit from anti-CTLA4 therapy, ORR of 30 and 32.5 %, respectively. This dataset has led to the FDA approval of single-agent nivolumab in ipilimumab-refractory metastatic melanoma patients in December 2014.

The studies reviewed above are briefly summarized in Table 2.

**Table 2:** Recent checkpoint inhibitor study results in melanoma

Study	Phase of study	Prior Tx	Agent	ORR (%)	PFS	OS
[25]	I	Yes/no	Pembrolizumab	31–51	>7 months	NA
[5]	I	Yes	Pembrolizumab	26	37–45 % at 6 months	58–63 % at 1 year
[26]	II	Yes	Pembrolizumab	21–25	34–38 % at 6 months	NA
			Chemotherapy	4	16 % at 6 months	
[27] [28]	I/II	Yes/no	Pembrolizumab	33	35 % at 1 year	23 months 40 % at 3 years
[29] [30]	III	No	Pembrolizumab	32.9–33.7	46.4-47.3 % at 6 months	55 % at 2 years
			Ipilimumab	11.9	26.5 % at 6 months	43 % at 2 years
[6]	I	Yes	Nivolumab	31	27 % at 2 years	16.8 months 43 % at 2 years
[32]	III	No	Nivolumab	40.0	5.1 months	72.9 % at 1 year
			DTIC	13.9	2.2 months	42.1 % at 1 year
[33]	III	Yes	Nivolumab	31.7	48 % at 6 months	NA
			Chemotherapy	10.6	34 % at 6 months	

*Prior Tx* patients with prior treatment for metastatic melanoma included. Yes/No indicates a mixed population of treatment naïve and pre-treated patients

## Combination of checkpoint inhibitors

The success of CTLA-4 inhibition and PD-1/PD-L1 inhibition in the treatment of cancer has lead to greater appreciation of the complexity of tumor microenvironment and the various interacting components that may present as unique opportunities for manipulation to control cancer. Conventional activated CD4+ and CD8+ T cells expressed CTLA-4 on the surfaces following induction [34, 35]. CTLA-4 binds to B7.1 (CD80) and B7.2 (CD86) on antigen presenting cells (APCs), where it competes with costimulatory receptor CD28. Binding of CTLA-4 to CD80/CD86 reduces CD28-dependent costimulation. CTLA-4 also mediates direct inhibitory effects on the MHC-TCR pathway by impairing TCR signaling [36]. Furthermore, CTLA-4 is constitutively expressed on CD4+CD25+FOXP3+ regulatory T cells and plays a role in their suppressive functions [37–39]. Programmed cell death protein-1 (PD-1; also known as CD27) is also a coinhibitory CD28-family molecule [35]. While CTLA-4 functions in the early phase of naïve T cell activation, PD-1 is mainly active in the late phase by inducing exhaustion in effector T cells. PD-1 is expressed on activated T cells, T regs [40], activated B cells, NK cells, and monocytes. It binds to PD-L1 (programmed death ligand-1, B7-H1) and PD-L2 (programmed death ligand-2, B7-DC) on APCs. PD-1 binding results in decreased TCR signaling [36]. Tumor cells utilize the PD-1-PD-L1/2 pathway to evade immune surveillance [41]. The observation that PD-1 inhibition is active in CTLA-4 inhibitor refractory patients confirms the complementary effects of dual checkpoint inhibition [5, 33].

In a randomized double-blind 3-arm study, treatment naive unresectable stage III or IV melanoma patients (n = 945) were randomized in a 1:1:1 ratio to nivolumab 3 mg/kg every 2 weeks, nivolumab 1 mg/kg every 3 weeks plus ipilimumab 3 mg/kg every 3 weeks for four doses followed by nivolumab 3 mg/kg every 2 weeks for cycle three and beyond, or ipilimumab 3 mg/kg every 3 weeks for four doses [42]. The median PFS was 11.5 months in the combination group compared with 2.9 months in the ipilimumab group (HR 0.42; 99.5 % CI, 0.31–0.57; p < 0.001) and 6.9 months in the nivolumab group (HR for the comparison with ipilimumab, 0.57; 99.5 % CI, 0.43–0.76; p < 0.001). While median PFS was similar between the combination group and the nivolumab group in patients with tumors positive for the PD-1 ligand (PD-L1) at 14.0 months, median PFS was longer with the combination therapy than with nivolumab alone in patients with PD-L1–negative tumors (11.2 versus 5.3 months). The activity of nivolumab-ipilimumab combination was similar in patients with and without BRAF mutation. ORR were 43.7 % in the nivolumab group, 57.6 % in the combination group, and 19.0 % in the ipilimumab group. The percentage of CR was higher in the combination group (11.5 %) than in either the nivolumab group (8.9 %) or the ipilimumab group (2.2 %). More treatment related AEs of grade 3 or 4 occurred in the combination group (55.0 %) compared with those of the nivolumab group (16.3 %) and the ipilimumab group (27.3 %). Discontinuation of treatment due to AEs also occurred more frequently in the combination group (36.4 %) compared with nivolumab group (7.7 %) and ipilimumab group (14.8 %). While one study-drug related death was reported in the nivolumab group (neutropenia) and one in the ipilimumab group (cardiac arrest), none was reported in the combination group. The study results were updated after more than18 months of follow-up [43]. Median PFS continued to be significantly longer for combination group (11.5 months) and nivolumab group (6.9 months) compared with ipilimumab group (2.9 months) (p < 0.001). Median duration of response for combination group responders has not been reached compared with 22.3 months for the nivolumab responders and 14.4 months for the ipilimumab responders.

The improvement in tumor response with combination CTLA-4 and PD-1 inhibition compared with CTLA-4 inhibition alone was also observed in another randomized double-blind trial [44]. Treatment naïve metastatic melanoma patients (n = 142) were randomly assigned in a 2:1 ratio to ipilimumab 3 mg/kg and nivolumab 1 mg/kg or placebo once every 3 weeks for four doses, followed by nivolumab 3 mg/kg or placebo every 2 weeks. Among patients with BRAF wild-type tumors, ORR was 61 % in the combination group versus 11 % in the monotherapy (p < 0.001). There were 16 patients (22 %) with CR in the combination group and none in the monotherapy group. Similar findings were also observed among patients with BRAF mutation–positive tumors with ORR of 52 % for those receiving combination therapy and CR rate of 22 %. Similar to the observation in the other randomized trial, the combination therapy is associated with more frequent grade 3 or 4 drug-related AEs compared with monotherapy group, 54 versus 24 %. The most common grade 3 or 4 AEs associated with the combination therapy were colitis (17 %), diarrhea (11 %), and an elevated alanine aminotransferase level (11 %). However, there was no significant difference in response rates between patients whose pretreatment tumors were defined as PD-L1–positive and those whose tumors were PD-L1–negative. Despite the significant clinical efficacy with combination of nivolumab and ipilimumab demonstrated in these initial studies, caution was raised for further studies to clarify the various patient subsets with optimal upfront therapy [45].

The impressive efficacy with dual CTLA-4 and PD-1 inhibition was also reported in the KEYNOTE-029 expansion trial testing pembrolizumab and ipilimumab combination [46]. Treatment naïve metastatic melanoma patients (n = 153) received pembrolizumab 2 mg/kg every 3 weeks plus ipilimumab 1 mg/kg every 3 weeks for 4 doses followed by pembrolizumab 2 mg/kg every 3 weeks until intolerable toxicity, progression, or 24 months. Grade 3–4 treatment related AEs were observed in 41 patients (38 %). ORR by central review was 57 % with 10 % CR and 47 % partial responses (PR). PFS at 6 months was 70 %.

The studies reviewed above are briefly summarized in Table 3.

**Table 3:** Recent combination checkpoint inhibitor study results in melanoma

Study	Phase of study	Prior Tx	Agents	ORR	PFS
[42] [43]	III	No	Ipilimumab	19.0	2.9 months
			Nivolumab	43.7	6.9 months
			Nivolumab + ipilimumab	57.6	11.5 months
[44]	III	No	Nivolumab + ipilimumab	52–61	8.5 months (BRAF mutant) Not reached (BRAF wildtype)
			Ipilimumab	11	2.7 months (BRAF mutant) 4.4 months (BRAF wildtype)
[46]	II	No	Pembrolizumab + ipilimumab	57	70 % at 6 months

## Oncolytic virus

Another promising novel therapy of melanoma is the recently approved genetically modified oncolytic virus for intralesional injection of cutaneous, subcutaneous, and nodal metastatic melanoma patients who progressed after surgery. Oncolytic virus preferentially replicates in tumor cells which results in tumor cell lysis. Following cell lysis, tumor-associated antigens and danger-associated molecules were released to promote anti-tumor immune responses as well as release of new viral particles for infecting nearby viable tumor cells. In the case of the recently approved talimogene laherparepvec (T-VEC), anti-tumor immune response following oncolysis is putatively enhanced by viral expression of GM-CSF which was inserted into the T-VEC genome. T-VEC is a Herpes simplex virus type-1 with several key genetic modifications. Deletion of ICP34.5 gene results in enhanced viral replication in cancer cells [47]. Deletion of ICP47 enhances antigen presentation and maintains cell surface MHC-I-antigen expression on infected cancer cells [48, 49]. Further improving the immunogenicity of T-VEC, 2 copies of the human GM-CSF gene were incorporated into the deleted ICP34.5 genome location [50].

In a phase I study, T-VEC has been to shown to replicate intratumorally and express GM-CSF with acceptable safety profile [51]. In phase II study, ORR of 26 % (n = 13) was observed in 50 stage IIIc and IV melanoma patients with 8 CRs and 5 PRs [52]. Responses were observed in both injected and uninjected lesions, including visceral lesions. In a phase III multicenter trial, 436 patients with unresectable stage IIIB-IV melanoma patients were randomized at a 2:1 ratio to intralesional T-VEC (n = 295) or subcutaneous GM-CSF (n = 141) [53]. Durable response rates (DRR; responses lasting 6 months or more) was significantly higher in the T-VEC group (16.3 %; 95 % CI 12.1–20.5 %) compared with GM-CSF control group (2.1 %; 95 % CI 0–4.5 %; odds ratio, 8.9; p = 0.001). ORR in the T-VEC arm was higher at 26.4 % compared with 5.7 % in the control arm. Although there was a trend for longer median OS in the T-VEC arm at 23.3 months compared with 18.9 months in the control arm, it was not significant (p = 0.051). The most common AEs with T-VEC were fatigue, chills, and pyrexia. The most common grade 3 or 4 AE was cellulitis (2.1 %). This dataset led to the FDA approval of T-VEC for intratumoral injection of cutaneous, subcutaneous, and nodal metastatic melanoma. To further elucidate the immunologic basis of its mechanism of action, a post hoc analysis of the phase II study (n = 50) was performed [54]. Reduction of tumor burden ≥30 % of uninjected non-visceral lesions was observed in 11 of 23 patients (47.8 %) and ≥30 % reduction in the total burden of visceral lesions was observed in 2 of 12 patients (16.7 %).

## Multimodal combination therapy

With the promising activity of both RAS-RAF-MAPK pathway inhibition and checkpoint inhibition in melanoma treatment, combination of both modes of therapy has been attempted. BRAF inhibition can have immunosensitization effects through increased antigen presentation, [55–57]. Antigen-specific T cell recognition [56, 58], homing of immune effector cell to the tumors [57, 59, 60] and improved T cell effector functions [61, 62]. MEK inhibition can also have immunosensitizing effect via up-regulation of tumor antigen expression and presentation [55, 63]. Paradoxically, MEK inhibitor can also dampen immune effector functions since impaired T cell proliferation and functions with MEK inhibition have been demonstrated in vitro [55, 64].

However, significant liver toxicity resulting in trial termination was observed in the first clinical trial of vemurafenib plus ipilimumab [65]. The first cohort of six patients received a run-in period of 1 month of vemurafenib (960 mg BID) only, followed by ipilimumab (3 mg/kg every 3 weeks for four doses) and concurrent BID doses of vemurafenib. Grade 3 elevations in aminotransferase levels developed in four patients following the first infusion of ipilimumab plus vemurafenib. The first 4 of 6 patients enrolled in the second cohort received a lower dose of vemurafenib (720 mg BID) plus the full dose of ipilimumab. Elevations in aminotransferase levels (grade 3 in two patients and grade 2 in one patient) developed within 3 weeks after starting ipilimumab. The remaining two patients in the second cohort received vemurafenib alone.

In a study combining dabrafenib with or without trametinib and ipilimumab at doses of dabrafenib 100 mg BID, trametinib 1 mg QD and ipilimumab 3 mg/kg every 3 weeks for 4 doses, grade 3 colitis complicated by perforation was observed in 2 of 7 advanced melanoma patients [66]. The enrollment to the triple combination arm was stopped with ongoing enrollment to the dabrafenib and ipilimumab combination arm. Another ongoing phase I trial is evaluating the combination of an anti-PD-L1 antibody MEDI4736 (durvolumab) at 10 mg/kg every 2 weeks plus dabrafenib 150 mg BID and trametinib 2 mg QD [67]. Patients were stratified by BRAF mutation status into three different cohorts. BRAF-mutant patients received the triple combination and BRAF wild-type patients received durvolumab plus trametinib or sequential trametinib then durvolumab. In the BRAF mutant cohort, treatment with the triple combination resulted in an ORR of 69 %, and DCR of 100 %. In the BRAF WT cohorts, ORR and DCR were 21 and 79 %, respectively, in the combination group and 13 and 80 %, respectively, in the sequential group. There was no significant increase in immune related AEs reported to date.

T-VEC and ipilimumab combination was also tested in an open-label, multicenter phase Ib study [68]. Patients (n = 19) with unresectable stage IIIB-IVM1c melanoma with injectable cutaneous, subcutaneous, or nodal lesion and without prior systemic therapy were enrolled. Intratumoral T-VEC as administered according to approved dosages and schedule. Beginning in week 6, ipilimumab (3 mg/kg) was administered every 3 weeks for four infusions. One patient received one dose of T-VEC and withdrew consent. The efficacy analysis included 18 patients. Five patients (26.3 %) reported grade 3 or higher treatment-related AEs, with three patients (15.8 %) attributable to T-VEC and four patients (21.1 %) attributable to ipilimumab. ORR was 50 % with four patients having confirmed CR (22 %). Median time to response was 5.3 months (range, 2.6–8.1 months). DRR was 44 %. Responses were seen in both injected and uninjected lesions. Among the 35 injected lesions, 26 (74 %) regressed ≥50 % and 11 (31 %) regressed completely. Among the 23 uninjected measurable lesions, 12 (52 %) regressed ≥50 %, and nine (39 %) regressed completely. Regression of uninjected non-visceral lesions was observed in five of 10 (50 %) measurable visceral lesions with ≥50 % regression.

## Promising novel agents under development

### Glembatumumab vedotin

The human 560-amino-acid type I glycoprotein NMB (gpNMB) is an intracellular transmembrane protein that transits the cell surface with homology to pMEL-17, a melanocyte specific marker [69, 70]. It is over-expressed in melanoma [70]. Glembatumumab vedotin (CDX-011 or CR011-vcMMAE) was produced by covalently linking a fully human IGG2 monoclonal antibody against gpNMB (CR011) to monomethyl auristatin E (MMAE), a potent mitotic spindle formation inhibitor [71–73]. It is designed to deliver the MMAE payload upon binding to gpNMB on the tumor cells and releasing free MMAE following lysosomal internalization and proteolytic cleavage of the valine–citrulline linker. Cell death ensues after microtubule inhibition by the free MMAE. Glembatumumab vedotin was active against melanoma cell lines expressing gpNMB in vitro and in pre-clinical model [70, 74]. A phase I/II study was conducted to assess the safety and activity of glembatumumab vedotin in patients with unresectable stage III or stage IV melanoma [75]. Enrolled patients (n = 117) received glembatumumab vedotin at 3 dosing schedules: every 3 weeks (schedule 1, n = 79), 2 of 3 weeks (schedule 2, n = 15), and weekly (schedule 3, n = 23) at escalating dosages. Grade 3/4 treatment-related AEs occurring in ≥2 patients were rash, neutropenia, fatigue, neuropathy, arthralgia, myalgia, and diarrhea. Three treatment-related deaths (resulting from pneumococcal sepsis, toxic epidermal necrolysis, and renal failure) occurred at doses exceeding the MTDs. In the schedule 1 phase II expansion cohort (n = 34), there were five patients (15 %) with PR and 8 (24 %) with stable disease (SD) for 6 months. ORR was 2 of 6 (33 %) for schedule 2 MTD and 3 of 12 (25 %) for the schedule 3 MTD.

### Pv-10

PV-10 is a sterile preparation of 10 % solution of rose bengal disodium (RB) in 0.9 % saline for intralesional injection into tumors [76]. RB has been used as intravenous diagnostic dye for liver function and as topical solution for ophthalmic conditions [77, 78]. Following intratumor injection, PV-10 accumulates in tumor lysosomes resulting in rapid lysis of tumor cells [79]. The lysis of tumor cells following PV-10 injection also induces tumor-specific T cell–mediated immune response with regression of uninjected lesions [80, 81]. In a phase 1 study, 8 patients with dermal and/or subcutaneous metastatic melanoma were enrolled [81]. CR was observed in injected and uninjected lesions in 4 of the 8 patients. All 8 patients exhibited partial to complete regression of the injected lesion. Best overall response rate (BORR) at 12 weeks is 55 %. Of the 6 enrolled patients with history of prior ipilimumab, PD-1 inhibitor, and/or vemurafenib trestments, 4 of 6 exhibited CR of the injected and uninjected lesions. In a subsequent multicenter phase II study, 62 stage III and IV treatment-refractory patients with at least 1 cutaneous or subcutaneous lesion were enrolled [82]. Intralesional (IL) injection of PV-10 at 0.5 ml per cm^3^ lesion volume was administered into each measurable lesion up to 20 study lesions. Treatments were repeated at weeks 8, 12, and 16 for new non-target lesions or existing target or non-target lesions not exhibiting complete response. BORR of target lesions was 51 % with 26 % CR and 25 % PR. DCR was achieved in 69 % of patients. Median time to response was 1.9 months. The median duration of response was 4.0 months. On analysis of lesion-specific responses of the 491 target lesions, CR, PR and SD were 53, 5, and 12 %, respectively. 26 % of 42 patients with designated bystander lesions experienced complete regression of the uninjected bystander lesions. Treatment was well-tolerated with predominantly mild to moderate locoregional or injection site-related AEs.

## Conclusion

The treatment paradigm for the management of advanced metastatic melanoma patients continues to shift with the rapid development of effective agents and combination therapy. With the many available active anti-melanoma agents, the optimal treatment algorithm for various subsets of patients, e.g. BRAF mutants, NRAS mutants, PD-1 expressing, has yet to be determined. With further understanding of key pathway molecules in melanoma oncogenesis, elucidation of components of tumor microenvironment, fine-tuning of drivers in the host-tumor immune relationship, and introduction of novel anti-tumor agents [83, 84], the outcome for metastatic melanoma will continue to improve.
